# Menopausal hormone therapy and risk of dementia: health insurance database in South Korea-based retrospective cohort study

**DOI:** 10.3389/fnagi.2023.1213481

**Published:** 2023-09-07

**Authors:** Jin-Sung Yuk, Jin San Lee, Joong Hyun Park

**Affiliations:** ^1^Department of Obstetrics and Gynecology, Sanggye Paik Hospital, School of Medicine, Inje University, Seoul, Republic of Korea; ^2^Department of Neurology, Kyung Hee University Hospital, Kyung Hee University College of Medicine, Seoul, Republic of Korea; ^3^Department of Neurology, Sanggye Paik Hospital, School of Medicine, Inje University, Seoul, Republic of Korea

**Keywords:** dementia, women, menopausal hormone therapy, menopause, tibolone

## Abstract

**Introduction:**

Menopausal hormone therapy (MHT) is used to alleviate the symptoms associated with menopause, despite the lack of recommendations for MHT in preventing dementia. Recent nationwide studies have explored the association between MHT and dementia risk, but the findings remain limited. This study aims to investigate the association between MHT and the incidence of Alzheimer’s disease (AD) and non-AD dementia using national population data from Korea.

**Methods:**

We conducted a retrospective study using data from the National Health Insurance Service in Korea between January 1, 2002, and December 31, 2019. Women over 40 years were eligible for this study and classified into the MHT or non-MHT groups. The MHT group consisted of women who used Tibolone (TIB), combined estrogen plus progestin by the manufacturer (CEPM), estrogen, combined estrogen plus progestin by a physician (CEPP), and transdermal estrogen during menopause. We compared the risk of dementia between the MHT and non-MHT groups.

**Results:**

The study included 1,399,256 patients, of whom 387,477 were in the MHT group, and 1,011,779 were in the non-MHT group. The median duration of MHT was 23 months (range: 10–55 months). After adjusting for available confounders, we found that different types of MHT had varying effects on the occurrence of dementia. TIB (HR 1.041, 95% confidence interval (CI) 1.01–1.072) and oral estrogen alone (HR 1.081, 95% CI 1.03–1.134) were associated with a higher risk of AD dementia. In contrast, there was no difference in the risk of AD dementia by CEPM (HR 0.975, 95% CI 0.93–1.019), CEPP (HR 1.131, 95% CI 0.997–1.283), and transdermal estrogen (HR 0.989, 95% CI 0.757–1.292) use. The use of TIB, CEPM, and oral estrogen alone increased the risk of non-AD dementia (HR 1.335, 95% CI 1.303–1.368; HR 1.25, 95% CI 1.21–1.292; and HR 1.128, 95% CI 1.079–1.179; respectively), but there was no risk of non-AD dementia in the other MHT groups (CEPP and topical estrogen).

**Conclusion:**

Our findings indicate that MHT has varying effects on the incidence of AD and non-AD dementia. Specifically, TIB, CEPM, and oral estrogen alone increase the risk of non-AD dementia, while transdermal estrogen is not associated with dementia risk. It is essential to consider the type of MHT used when assessing the risk of dementia in women.

## 1. Introduction

Dementia is characterized by a decline in cognition involving one or more of the following cognitive domains: memory and learning, language, executive function, complex attention, or social cognition (McKhann et al., 2011). The number of people with dementia worldwide was estimated at 47.47 million in 2015 and is expected to reach 135.46 million in 2050. Due to the increasing number of older adults, dementia and age-related cognitive disorders present a significant challenge for health services and an increasing global problem (Ferri et al., 2005; Livingston et al., 2020; Tam et al., 2021).

This trend seems more rapid in South Korea (hereafter Korea). Based on the government-led data, it is estimated that the number of people aged ≥65 years will reach 8.53 million in 2021 and is expected to exceed 12.98 million by 2030 and 19 million (accounting for 39.8% of the older adult population) by 2050 (Jang et al., 2021; Kim J.H. et al., 2022). Additionally, the burden of dementia in Korea is an important issue and is expected to increase considerably, and the annual management cost per patient was approximately 20.72 million won in 2019 (Kong and Park, 2022). The cost of managing dementia at the national level is estimated to be 16.5 trillion won (approximately 0.86% of GDP), which is expected to increase to 63 trillion won by 2040 (Park et al., 2013; Kong and Park, 2022).

Most studies have shown that women have a higher prevalence of dementia than men. This trend, which is generally explained by differences in sex hormones, education, and life expectancy, becomes more evident as age increases, because the prevalence of Alzheimer’s disease (AD) is higher in women aged >80 years than in men (Ferri et al., 2005; McKhann et al., 2011). These demographic characteristics suggest an anticipated increase in the burden of dementia in women. Identifying women at a higher risk of dementia and correcting modifiable risk factors are essential (Derreberry and Holroyd, 2019).

Menopausal hormone therapy (MHT), which is prescribed for postmenopausal women with moderate to severe menopausal vasomotor symptoms, is not recommended to prevent dementia (Lee et al., 2020; Pinkerton, 2020). Although some epidemiological and observational studies on MHT in aging women support the positive effects of estrogen on cognitive function (Imtiaz et al., 2017), data from the Women’s Health Initiative and extensive research have shown that estrogen replacement does not protect against dementia and may increase its risk (Shumaker et al., 2004; McCarrey and Resnick, 2015; Pinkerton, 2020). Tibolone (TIB) treatment, as an alternative to conventional hormone replacement therapy, has estrogenic, progestogenic, and androgenic effects (Huang et al., 2010). Although there are many protective actions for the nervous system, TIB is contraindicated in women with a history of breast cancer and may increase the risk of stroke (Modelska and Cummings, 2002; Formoso et al., 2016). Although the number of participants was small, a recent nationwide case–control study showed that TIB treatment increased the risk of AD in women older than 60 years (Savolainen-Peltonen et al., 2019). Another nationwide study using Korean claims data suggested that TIB treatment did not significantly affect dementia risk aged in patients aged 50–80 years (Han et al., 2021). However, this study did not include possible confounders, such as the duration and initiation age of MHT. Furthermore, it did not adjust for various essential risk factors for dementia and gynecological factors, such as menstrual history, excessive alcohol intake, and physical activity.

Although recent nationwide studies have been conducted on the relationship between MHT and the risk of dementia, findings on this issue are limited. This study aimed to explore the risk of dementia and MHT using the Health Insurance Database of South Korea.

## 2. Materials and methods

### 2.1. Database

We conducted a retrospective study based on the national population by investigating data from the National Health Insurance Service (NHIS) in Korea from January 1, 2002, to December 31, 2019. Korea’s health insurance system was integrated into a single system operated by the National Health Insurance Corporation in 2000 (Seong et al., 2017; Noh et al., 2019). Since the National Health Insurance covers most people living in Korea (about 51 million people), most disease health insurance information (age, sex, diagnostic name, surgical name, and prescription) can be checked, except for procedures not covered by insurance, such as plastic surgery (Kim et al., 2014; Noh et al., 2019). In addition, the NHIS recommends that employees and insured people aged ≥40 years undergo free cardiovascular health checkups every other year and that physical labor workers should be examined every year (Seong et al., 2017). Therefore, the NHIS provides additional health examination data for these people. Similarly, since the National Cancer Screening Program was introduced in 1999, all individuals at a certain age are given free gastric, liver, colon, breast, and cervical cancer screening every two years for all women aged >30 years (Yoo, 2008; Yun et al., 2020; Bolormaa et al., 2022). The NHIS provides additional self-survey data on history and cancer examination results. The International Classification of Diseases, 10th revision (ICD-10) was used to record the diagnosis name, and surgery and procedures were confirmed using the Korean Health Insurance Medical Care Expenses (2012, 2016, and 2019 versions) (Seong et al., 2017).

### 2.2. Selection of participants

**Figure 1:**
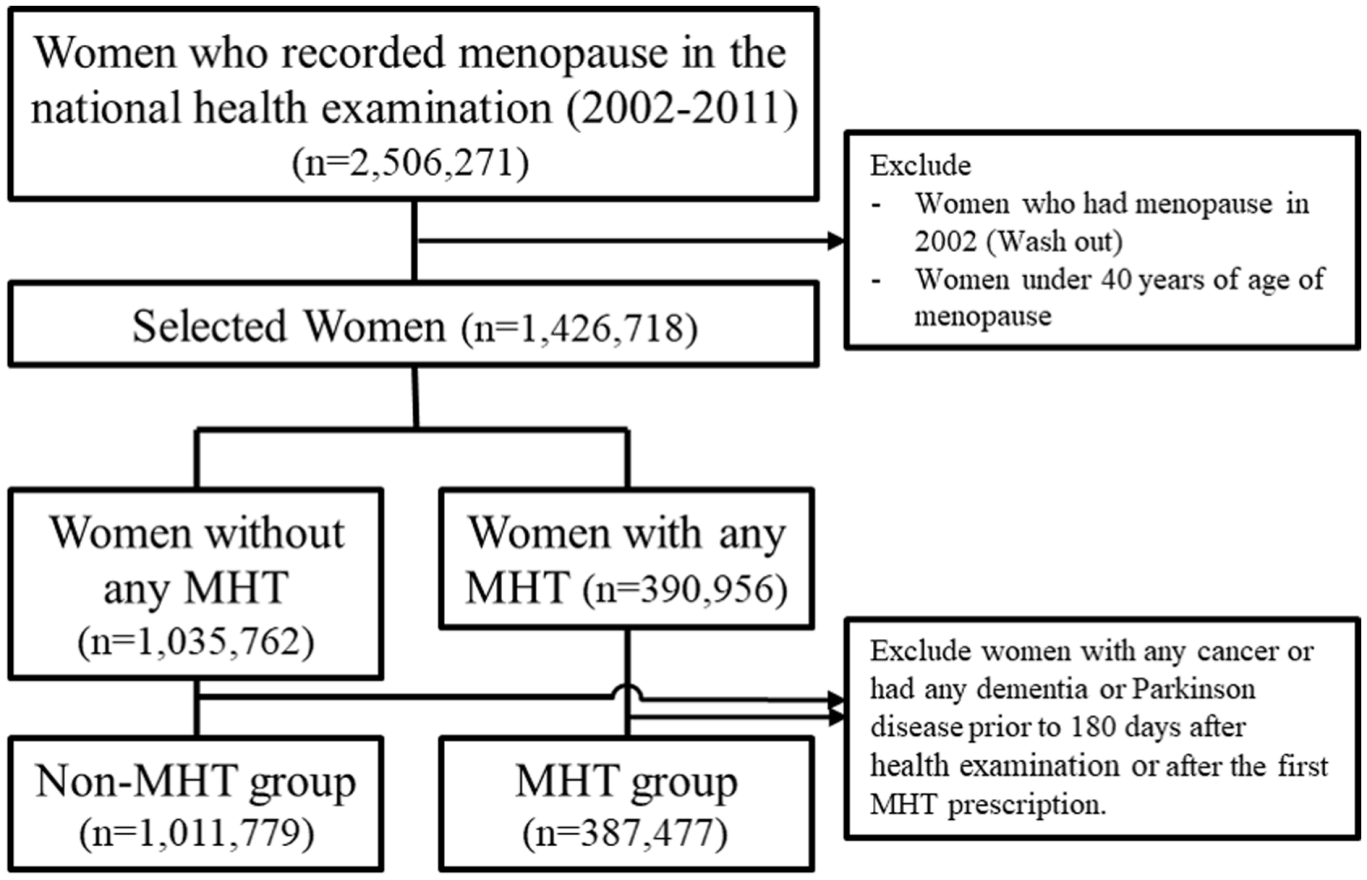
Flowchart for the selection of case–control according to MHT in Korea National Health Insurance Data, 2002–2019. MHT, menopausal hormone therapy.

Using a database of clinical records from 2002 to 2011, only women aged >40 years were eligible for this study, and participants were recruited into the MHT and non-MHT groups. The MHT group included patients prescribed at least one MHT between 2002 and 2011, while the non-MHT group were those who underwent a national health examination at least once between January 1, 2002, and December 31, 2011, excluding those who received MHT between 2002 and 2019. Participants with previous diagnostic codes for cancer (any Cxx), dementia (F00–03, G30–31), or Parkinson’s disease (G20) before the 180th day from the start date of the study were excluded. Women diagnosed with menopause in 2002 and those aged <40 years were also excluded (Figure 1).

### 2.3. Outcome

The term ‘AD dementia’ used in this study is a clinical diagnostic term of AD dementia based on the ICD-10 code. AD dementia was defined based on diagnostic ICD-10 codes (F00 or G30) and non-AD dementia based on the codes (F01, F02, F03, G231, or G31), among patients who had visited three or more medical institutions. Total dementia (TD) was defined when the above two conditions are satisfied.

### 2.4. Variables

In this study, MHT during menopause refers to the use of TIB, combined estrogen plus progestin by the manufacturer (CEPM), estrogen, combined estrogen plus progestin by a physician (CEPP), and transdermal estrogen. A detailed list of the MHTs is provided in Supplementary Table S1. If it was confirmed that two or more MHTs were sequentially used, the last MHT used for more than 6 months was defined as the woman’s treatment. The reference date for collection of data such as age, parity, age at menarche, age at menopause, body mass index (BMI), socioeconomic status (SES), region of the clinic, Charlson Comorbidity Index (CCI), period from menopause to inclusion date, smoking status, alcohol consumption status, and physical exercise was the date of participation in the study. MHT initiation date was defined as the date on which the first MHT was prescribed. On the other hand, the examination date in the institutions was defined as the treatment start date for the non-MHT group. If the start date of treatment is not confirmed and only the year of examination is recorded, June 30 of the year of examination is defined as the initiation date. The date of death (or December 31, 2019) was defined as the last day without other events. BMI in this study was evaluated based on the criteria presented from an Asia-Pacific perspective (WHO expert consultation, 2004). Patients on Medicaid for medical insurance were categorized as having an SES. An urban area is defined as an area in which the administrative district is a large city (WHO expert consultation, 2004). We calculated CCI using the diagnosis codes evaluated at the medical institutions visited from 1 year before the date of participation to the date of participation in the study (Quan et al., 2011). Current smokers were defined as those who smoked during our investigation. Never smokers and past smokers were defined as those who reported having smoked in their lifetime and those who reported having smoked but did not smoke during the study. Alcohol history was classified according to the weekly drinking frequency (None, ~2/week, 3 ~ 6/week, and daily). Physical exercise was classified according to the number of exercises performed for 30 min or more per week.

### 2.5. Statistical analyses

A two-sided test was performed for all statistics, and a value of *p* <0.05 was considered statistically significant. All continuous data are presented as median [25th and 75th percentile], and categorical data are expressed as total number (%). Cox proportional hazard regression analysis, with or without adjustment for confounders, was used to analyze the associations between the variables and dementia. The pairwise deletion method was used when values were missing. All statistical analyses were carried out using SAS Enterprise Guide 6.1 (SAS Institute Inc.). To confirm the sensitivity analysis for evaluating the robustness of these results, we analyzed only participants prescribed medications by an obstetrics and gynecology specialist.

### 2.6. Ethics statement

This study was approved by the Institutional Review Board of the Sanggye Paik Hospital (approval number: SGPAIK-2020-08-002). Following the NHIS’s information protection policy, this study removed individual-identifying variables that could only be analyzed using virtual servers within the NHIS. Therefore, this study was conducted retrospectively using data obtained for clinical purposes, and the researcher could secure only the results. Furthermore, because it is impossible to specify any individual in the data, no possible harm can be caused. Moreover, this study did not require informed consent from the participants according to the Bioethics and Safety Act of South Korea.

## 3. Results

### 3.1. Baseline characteristics

Of the 2,506,271 women who recorded menopause in the national health examination during the study period (2002–2011), we excluded those who had menopause in 2002 (washout period) and those under 40 years old. Women with any cancer type, previous dementia, or Parkinsonian syndrome within 180 days after the health examination or after the first MHT prescription were also excluded. Finally, data from 1,399,256 patients were included in this study. Among them, 387,477 were classified into the MHT group and 1,011,779 into the non-MHT group (Figure 1).

The baseline characteristics of the women according to MHT exposure status are summarized in Table 1. The most frequently prescribed MHT was TIB (195,476; median age: 52 years), followed by CEPM (130,246; median age: 53 years), oral estrogen alone (53,143; median age: 54 years), CEPP (6,531; median age: 54 years), and transdermal estrogen (2,081; median age: 53 years). The number of patients with TD was 96,853 (9.6%) in the non-MHT group, 13,372 (6.8%) in the TIB group, 6,183 (4.7%) in the CEPM group, 3,696 (7%) in the oral estrogen alone group, 494 (7.6%) in the CEPP group, and 115 (5.5%) in the transdermal estrogen group.

**Table 1:** Baseline characteristics of women according to menopausal hormone exposure status at recruitment, Korea National Health Insurance Data, 2002–2019.

	Non-MHT	TIB	CEPM	Oral Estrogen	CEPP	Transdermal estrogen	Total
Number of women	1,011,779	195,476	130,246	53,143	6,531	2,081	1,399,256
Median age (years)	53 [50–57]	52 [50–56]	53 [49–57]	54 [51–59]	54 [51–59]	53 [50–58]	58 [52–64]
Median follow-up period (years)	11.5 [9.5–13.5]	12.8 [10.5–14.8]	11.9 [10.1–14.3]	13.9 [11.4–15.7]	13.8 [11.1–15.9]	14.2 [12.2–15.7]	11.5 [9.5–13.5]
*Age at inclusion (years)*							
40 ~ 49	85,683 (8.5)	34,331 (17.6)	29,826 (22.9)	13,831 (26)	1,143 (17.5)	439 (21.1)	165,253 (11.8)
50 ~ 59	493,744 (48.8)	128,026 (65.5)	86,702 (66.6)	29,760 (56)	3,850 (58.9)	1,271 (61.1)	743,353 (53.1)
60 ~ 69	304,315 (34.4)	29,443 (15.4)	12,679 (9.8)	7,873 (15.3)	1,345 (21.2)	336 (16.4)	355,991 (28.2)
70~	128,037 (12.7)	3,676 (1.9)	1,039 (0.8)	1,679 (3.2)	193 (3)	35 (1.7)	134,659 (9.6)
Median BMI (kg/m^2^)	24 [22.1–26.1]	23.5 [21.8–25.4]	23.1 [21.5–25]	23.7 [22–25.7]	23.3 [21.6–25.2]	23.7 [22–25.6]	23.8 [21.9–25.9]
*BMI (kg/m2)*							
<18.5	18,757 (1.9)	3,347 (1.7)	2,608 (2)	775 (1.5)	134 (2.1)	45 (2.2)	25,666 (1.9)
18.5–22.9	340,070 (34.3)	78,531 (40.6)	58,556 (45.3)	19,491 (37)	2,696 (41.6)	768 (37.2)	500,112 (36.3)
23–24.9	262,812 (26.5)	53,719 (27.7)	34,763 (26.9)	14,746 (28)	1,815 (28)	552 (26.7)	368,407 (26.8)
25–29.9	328,197 (33.1)	53,116 (27.4)	30,852 (23.9)	15,912 (30.2)	1,689 (26.1)	641 (31.1)	430,407 (31.3)
≥30	43,002 (4.3)	4,909 (2.5)	2,557 (2)	1,786 (3.4)	142 (2.2)	58 (2.8)	52,454 (3.8)
*SES*							
Mid ~ high SES	970,198 (95.9)	188,565 (96.5)	126,981 (97.5)	51,674 (97.2)	6,365 (97.5)	2,021 (97.1)	1,345,804 (96.2)
Low SES	41,581 (4.1)	6,911 (3.5)	3,265 (2.5)	1,469 (2.8)	166 (2.5)	60 (2.9)	53,452 (3.8)
*Region*							
Urban area	298,338 (29.5)	60,734 (31.1)	43,925 (33.7)	16,682 (31.4)	3,297 (50.5)	942 (45.3)	423,918 (30.3)
Rural area	713,441 (70.5)	134,742 (68.9)	86,321 (66.3)	36,461 (68.6)	3,234 (49.5)	1,139 (54.7)	975,338 (69.7)
*CCI*							
0	664,370 (65.7)	132,453 (67.8)	91,893 (70.6)	36,856 (69.4)	4,498 (68.9)	1,351 (64.9)	931,421 (66.6)
1	201,286 (19.9)	38,429 (19.7)	23,834 (18.3)	9,849 (18.5)	1,234 (18.9)	393 (18.9)	275,025 (19.7)
≥2	146,123 (14.4)	24,594 (12.6)	14,519 (11.1)	6,438 (12.1)	799 (12.2)	337 (16.2)	192,810 (13.8)
*Parity (years)*							
0 or not respond	165,454 (16.4)	30,289 (15.5)	16,242 (12.5)	11,153 (21)	1,285 (19.7)	455 (21.9)	224,878 (16.1)
1	60,098 (5.9)	17,054 (8.7)	13,578 (10.4)	4,091 (7.7)	487 (7.5)	167 (8)	95,475 (6.8)
2	664,541 (74.7)	130,455 (73.4)	90,873 (75.3)	32,657 (68.2)	4,091 (69.8)	1,251 (66.8)	923,868 (74.3)
≥3	121,686 (12)	17,678 (9)	9,553 (7.3)	5,242 (9.9)	668 (10.2)	208 (10)	155,035 (11.1)
*Age at menarche (years)*							
<13	157,857 (15.7)	28,371 (14.6)	18,522 (14.3)	9,752 (18.6)	1,212 (18.7)	387 (18.8)	216,101 (15.5)
≥13	849,189 (84.3)	165,588 (85.4)	110,982 (85.7)	42,709 (81.4)	5,264 (81.3)	1,669 (81.2)	1,175,401 (84.5)
*Age at menopause (years)*							
40–44	120,943 (12)	23,411 (12)	14,623 (11.2)	11,459 (21.6)	834 (12.8)	412 (19.8)	171,682 (12.3)
45–49	291,724 (28.8)	63,608 (32.5)	43,607 (33.5)	18,852 (35.5)	2,095 (32.1)	751 (36.1)	420,637 (30.1)
50–54	510,195 (55.3)	93,644 (51.8)	63,392 (52.1)	20,240 (40)	3,085 (51.3)	789 (40.4)	691,345 (53.9)
55-	88,917 (8.8)	14,813 (7.6)	8,624 (6.6)	2,592 (4.9)	517 (7.9)	129 (6.2)	115,592 (8.3)
*Smoking*							
Never	923,518 (96.4)	176,371 (93.7)	117,896 (93.5)	48,550 (95)	6,019 (95.7)	1,907 (96.1)	1,274,261 (95.7)
Past	9,755 (1)	3,223 (1.7)	2,320 (1.8)	713 (1.4)	82 (1.3)	32 (1.6)	16,125 (1.2)
Current	25,047 (2.6)	8,560 (4.5)	5,902 (4.7)	1,852 (3.6)	189 (3)	45 (2.3)	41,595 (3.1)
*Alcohol (per week)*							
None	820,873 (85.3)	146,881 (77.5)	96,196 (75.8)	41,050 (79.7)	5,242 (82.7)	1,658 (82.4)	1,111,900 (83.1)
~2/week	120,759 (12.6)	36,109 (19.1)	26,181 (20.6)	9,095 (17.7)	968 (15.3)	312 (15.5)	193,424 (14.5)
3 ~ 6/week	14,997 (1.6)	4,951 (2.6)	3,572 (2.8)	993 (1.9)	90 (1.4)	31 (1.5)	24,634 (1.9)
Daily	5,380 (0.6)	1,519 (0.8)	924 (0.7)	385 (0.7)	40 (0.6)	10 (0.5)	8,258 (0.6)
*Physical exercise (per week)*							
None	631,239 (65.5)	112,640 (59.5)	76,031 (59.9)	30,716 (59.7)	3,624 (57.2)	1,057 (52.8)	855,307 (63.8)
1 ~ 2	158,439 (16.4)	36,118 (19.1)	24,736 (19.5)	9,891 (19.2)	1,236 (19.5)	430 (21.5)	230,850 (17.2)
3 ~ 4	87,866 (9.1)	21,741 (11.5)	14,821 (11.7)	5,713 (11.1)	813 (12.8)	297 (14.8)	131,251 (9.8)
5 ~ 6	29,422 (3.1)	7,126 (3.8)	4,846 (3.8)	1,808 (3.5)	245 (3.9)	86 (4.3)	43,533 (3.2)
Daily	56,610 (5.9)	11,713 (6.2)	6,466 (5.1)	3,358 (6.5)	417 (6.6)	132 (6.6)	78,696 (5.9)
*Period from menopause to inclusion (years)*							
<5	396,090 (39.1)	113,704 (58.2)	88,755 (68.1)	27,783 (52.3)	3,347 (51.2)	1,055 (50.7)	630,734 (45.1)
5 ~ 9	212,604 (21)	44,936 (23)	25,830 (19.8)	13,287 (25)	1,568 (24)	546 (26.2)	298,771 (21.4)
10~	403,085 (39.8)	36,836 (18.8)	15,661 (12)	12,073 (22.7)	1,616 (24.7)	480 (23.1)	469,751 (33.6)

TIB, Tibolone; CEPM, Combined estrogen plus progestin by manufacturer; CEPP, Combined estrogen plus progestin by physician; BMI, Body mass index; CCI, Charlson comorbidity index; MHT, menopausal hormone therapy; SES, socioeconomic status. Data are expressed as the number (%) or median [25 percentile, 75 percentile].

### 3.2. Women receiving menopausal hormone therapy

Overall, the median duration of MHT was 23 months (range: 10–55 months). Only 23,145 (6%) women used hormone therapy for more than 10 years, whereas a shorter duration of MHT (shorter than 5 years) was observed in 297,801 (76.9%) women. According to the gynecology and non-gynecology specialties, physicians prescribed hormone therapy to 149,498 (38.6%) and 237,979 (61.4%) women, respectively. The detailed characteristics of the women receiving MHT in this study are shown in Table 2.

**Table 2:** Characteristics of women with menopausal hormone therapy, Korea National Health Insurance Data, 2002–2019.

MHT characteristics	TIB	CEPM	Oral Estrogen	CEPP	Transdermal estrogen	Total MHT
Median duration (months)	25 [11–58]	25 [11–58]	15 [9–40]	16 [9–35]	13 [8–24]	23 [10–55]
*Duration (years)*						
<5	147,530 (75.5)	98,633 (75.7)	43,962 (82.7)	5,694 (87.2)	1,982 (95.2)	297,801 (76.9)
5–9.9	35,208 (18)	24,201 (18.6)	6,380 (12)	648 (9.9)	94 (4.5)	66,531 (17.2)
≥10	12,738 (6.5)	7,412 (5.7)	2,801 (5.3)	189 (2.9)	5 (0.2)	23,145 (6)
*Duration of previous other MHT (years)*						
<5	190,078 (97.2)	128,128 (98.4)	52,353 (98.5)	5,439 (83.3)	2,055 (98.8)	378,053 (97.6)
5–9.9	4,823 (2.5)	1,938 (1.5)	709 (1.3)	811 (12.4)	25 (1.2)	8,306 (2.1)
≥10	575 (0.3)	180 (0.1)	81 (0.2)	281 (4.3)	1 (0)	1,118 (0.3)
*Last dosage of tibolone (per day)*						
1.25 mg	1,859 (1)					
2.5 mg	193,416 (99)					
over 5 mg	181 (0.1)					
*Prescribed specialty*						
Gynecology	65,811 (33.7)	59,661 (45.8)	21,946 (41.3)	1,566 (24)	514 (24.7)	149,498 (38.6)
Non-gynecology	129,665 (66.3)	70,585 (54.2)	31,197 (58.7)	4,965 (76)	1,567 (75.3)	237,979 (61.4)

MHT, menopausal hormone therapy; TIB, Tibolone; CEPM, Combined estrogen plus progestin by manufacturer; CEPP, Combined estrogen plus progestin by physician. Data are expressed as the number (%) or median [25 percentile, 75 percentile].

### 3.3. Association of reproductive factors with dementia risk in women receiving MHT

The hazard ratio for the risk of dementia according to reproductive factors are shown in Supplementary Table S2. In a dose-dependent manner, increase in the incidence of TD was significantly associated with later age at menarche, later age at inclusion, low SES, rural area, high CCI, parity, late menopausal age, smoking, and a long period from menopause to inclusion (years). Compared with short duration (5–9 years) from menopause to inclusion (hazard ratio (HR) 1.413, 95% confidence interval (CI) 1.379–1.448), long duration (10 years) was associated with a higher risk of TD (HR 2.165, 95% CI 2.103–2.228). Similar patterns were observed for both patients with AD and non-AD dementia (Supplementary Table S2). Supplementary Table S3 shows case/person-years of dementia according to the reproductive factors. Regardless of hormone type, there is an increased incidence of dementia with advanced age (60 ~ 69 years). Our findings also showed an increased risk of dementia with longer period from menopause to inclusion after menopause (<5 years versus >10 years).

### 3.4. Association MHT drug type and risk of dementia

**Figure 2:**
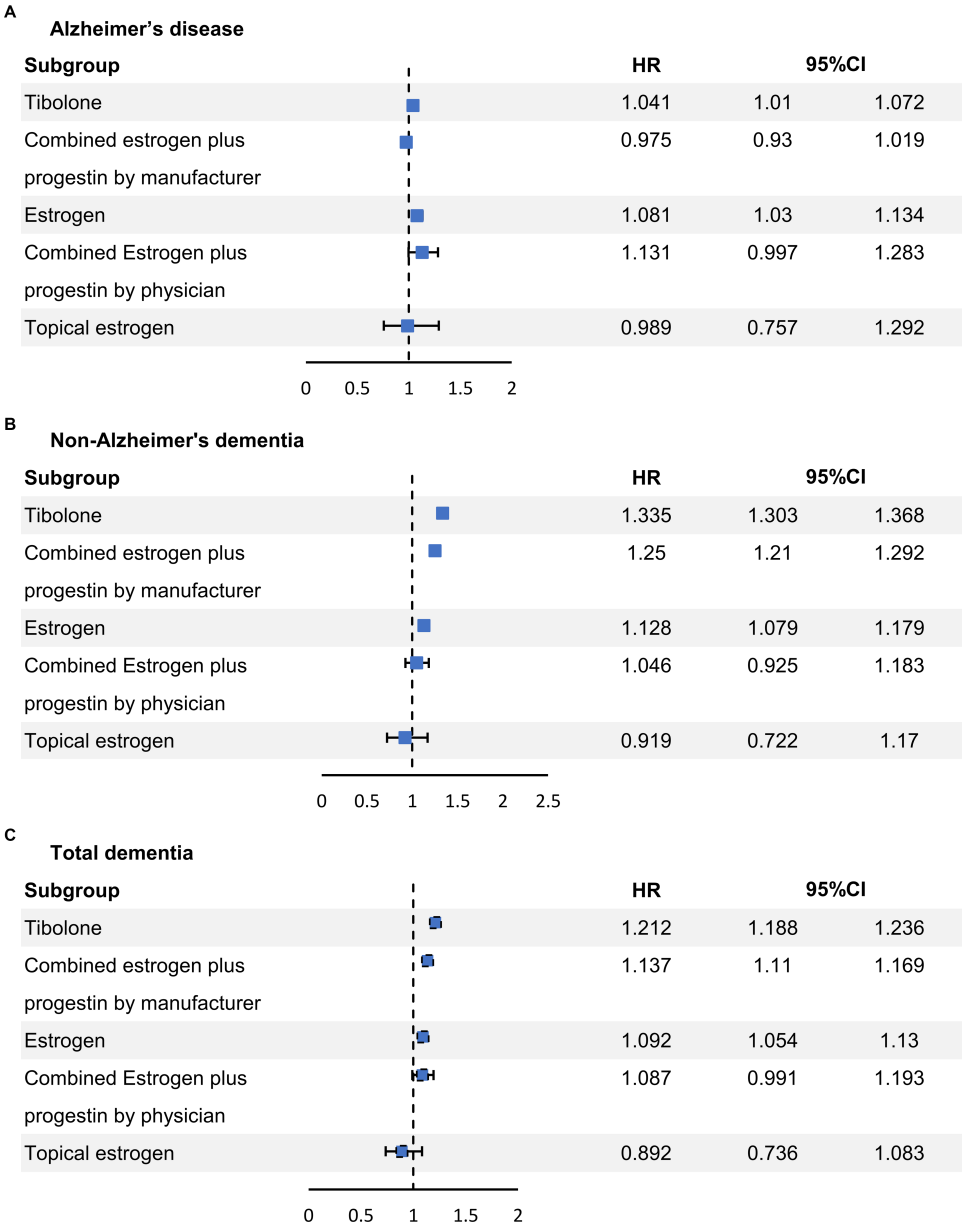
Conditional logistic regression analysis of the risk of each dementia phenotype based on the formulation of the MHT. MHT, menopausal hormone therapy. CI, confidence interval; HR, hazard ratio.

In Table 3, an unadjusted analysis of dementia incidence showed that the incidence of TD was higher in the non-MHT group (9.6%) than in the MHT group (8.6%). After adjusting for available confounders, such as age group, age at menarche, age at menopause, BMI, SES, region, CCI, parity, period from menopause to inclusion, smoking status, alcohol consumption status, and physical exercise, we found that different MHTs had varying effects on dementia occurrence. Figure 2 shows the risk of each dementia phenotype based on the MHT formulation. TIB (HR 1.041, 95% confidence interval (CI) 1.01–1.072) and oral estrogen alone (HR 1.081, 95% CI 1.03–1.134) were associated with a higher risk of AD dementia. In contrast, there was no difference in the risk of AD dementia by CEPM (HR 0.975, 95% CI 0.93–1.019), CEPP (HR 1.131, 95% CI 0.997–1.283), and transdermal estrogen (HR 0.989, 95% CI 0.757–1.292) use. The use of TIB, CEPM, and oral estrogen alone increased the risk of non-AD dementia (HR 1.335, 95% CI 1.303–1.368; HR 1.25, 95% CI 1.21–1.292; and HR 1.128, 95% CI 1.079–1.179, respectively), but there was no risk of non-AD dementia in the other MHT groups (CEPP and topical estrogen). Similarly, TD risk increased in users of TIB (HR 1.212, 95% CI 1.188–1.236), CEPM (HR 1.137, 95% CI 1.11–1.169), and oral estrogen alone (HR 1.092, 95% CI 1.054–1.13), but not in the other groups.

**Table 3:** Incidence of dementia according to tibolone exposure status at recruitment, Korea National Health Insurance Data, 2002–2019.

	Non-MHT	TIB	CEPM	Oral Estrogen	CEPP	Transdermal estrogen	Total
Median period from menopause to inclusion (years)	7 [2–14.5]	3 [1–8]	2 [0–6]	4 [1–9]	4.5 [1–9.5]	4.5 [1–9]	5.5 [2–12.5]
AD dementia							
Not present	948,489 (93.7)	189,732 (97.1)	127,885 (98.2)	51,205 (96.4)	6,263 (95.9)	2,018 (97)	1,325,592 (94.7)
Present	63,290 (6.3)	5,744 (2.9)	2,361 (1.8)	1,938 (3.6)	268 (4.1)	63 (3)	73,664 (5.3)
*Non-AD dementia*							
Not present	962,844 (95.2)	186,420 (95.4)	125,803 (96.6)	50,866 (95.7)	6,243 (95.6)	2,011 (96.6)	1,334,187 (95.3)
Present	48,935 (4.8)	9,056 (4.6)	4,443 (3.4)	2,277 (4.3)	288 (4.4)	70 (3.4)	65,069 (4.7)
*Total dementia*							
Not present	914,926 (90.4)	182,104 (93.2)	124,063 (95.3)	49,447 (93)	6,037 (92.4)	1,966 (94.5)	1,278,543 (91.4)
Present	96,853 (9.6)	13,372 (6.8)	6,183 (4.7)	3,696 (7)	494 (7.6)	115 (5.5)	120,713 (8.6)

MHT, menopausal hormone therapy; TIB, Tibolone; CEPM, Combined estrogen plus progestin by manufacturer; CEPP, Combined estrogen plus progestin by physician; AD, Alzheimer’s disease. Data are expressed as the number (%) or median [25 percentile, 75 percentile].

## 4. Discussion

We conducted a large cohort study of menopausal women undergoing MHT to determine the association between MHT use and the risk of dementia. Our study revealed that TIB and oral estrogen alone were associated with an increased risk of AD dementia. In contrast, CEPM, CEPP, and topical estrogen were not associated with an increased risk of AD dementia. In this analysis, the use of TIB, CEPM, and oral estrogen alone was found to be related to an increased risk of non-AD dementia and TD. However, topical estrogen was not associated with an increased risk of AD or non-AD dementia.

A few studies have reported neither the benefit nor the risk of MHT concerning AD, which begins later than 65 or more than 5 years after menopause. A recent study that assessed the risks of developing TD and AD in women exposed to different types of MHT for different durations also reported no increased risks of developing dementia overall (Vinogradova et al., 2021). Furthermore, a retrospective study using a 10-year claims dataset found that MHT was associated with a reduced risk of all neurodegenerative diseases, including TD and AD (Kim et al., 2021). However, most studies have suggested the potential and overall risks of MHT later in life concerning dementia, including AD. The most influential study, the Women’s Health Initiative Memory Study, reported a significantly increased TD risk for using conjugated equine estrogen (CEE) + medroxyprogesterone acetate and CEE alone (Shumaker et al., 2003). Similar results were noted in the Kronos Early Estrogen Prevention Cognitive and Affective Ancillary Study (Gleason et al., 2015). Early versus Late Intervention Trial with Estradiol-Cognitive Endpoints indicated no beneficial or harmful effects of MHT on dementia risk (Henderson, 2011; Henderson et al., 2016). Until recently, prescribing MHT purely for dementia prevention was not recommended in the absence of other clinical indications for MHT, such as vasomotor symptoms (hot flashes and night sweats) and sleep disturbances (Baber et al., 2016; Marjoribanks et al., 2017; North American Menopause Society, 2017). Our study, which adjusted for other dementia risk factors, also showed that hormone therapy increased the overall risk of dementia in elderly menopausal women. Moreover, our results (Supplementary Table S2) are consistent with the content of the current hormone therapy guidelines, explicitly emphasizing the potential benefits of hormone therapy for younger postmenopausal patients and those who commence treatment within 10 years of menopause onset.

The most important finding of this study was that MHT had different effects on the incidence of AD and non-AD dementia. In this study, TIB, CEPM, and estrogen-only use were associated with an increased risk of non-AD dementia, consequently increasing overall TD incidence. Excluding CEPP, most oral hormone therapies (TIB, CEPM, and estrogen) showed an increased tendency towards non-AD dementia occurrence. This is believed to be due to the accumulation of prothrombotic metabolites that may occur during the metabolic process of oral hormone therapy, leading to atherosclerotic effects, ischemia, and vascular cognitive decline (Henderson and Popat, 2011; Scarabin-Carre et al., 2012). Although TIB is known to show a cardiovascular protection effect through its metabolite (Del Rio et al., 2020), long-term use of TIB may also be associated with increased cardiovascular risks, such as CRP elevation and hyperthermia, which could lead to brain vessel wall injury (Formoso et al., 2016; Zhu et al., 2021). A similar mechanism can explain this as the analysis result that topical estrogen is not associated with non-AD dementia occurrence. In other words, topical estrogen, which has a hepatic first-pass effect, is not metabolized in the liver and does not produce various metabolites that can show adverse vascular effects (Simon, 2012; Beck et al., 2017; Oliver-Williams et al., 2019).

Previous studies have reported the neuroprotective effects of estrogen on the brain, cognition, and dementia (Yaffe et al., 2000; Carlson et al., 2001; Lui et al., 2003). Estrogen improves synapse formation in the hippocampus and increases choline acetyltransferase activity in the basal forebrain of rats (Aubele and Kritzer, 2011). Moreover, estrogen improves prefrontal lobe-dependent executive functioning in women (Krug et al., 2006). In addition to its cognitive effects, estrogen has neuroprotective effects, such as inhibiting amyloid-beta depositions in the brain, primarily forming neuropathological lesions in patients with AD (Sohrabji, 2005). However, an increased level of free estradiol may indicate atherothrombogenic stroke risk in older postmenopausal women (Henderson and Popat, 2011). Insulin resistance, dyslipidemia, and inflammation are the potential mediators of this association (Scarabin-Carre et al., 2012). Although the risk of ischemic stroke differs by age, recent studies of MHT on stroke incidence suggest that the risk of ischemic stroke increases with oral MHT use in women aged >60 years (Bushnell et al., 2014). Considering the incidence of all-cause dementia, most non-AD cases appear to be vascular dementia cases (O'Brien and Thomas, 2015). However, the precise mechanism by which MHT affects brain vessels and influences the incidence of ischemic stroke has not been established (Miller et al., 2003; Khalil, 2013). Considering vascular dementia and the increased risk of ischemic stroke due to MHT, our findings indicate that MHT specifically increases the risk of vascular dementia rather than AD dementia. Consequently, the incidence of overall dementia increased among postmenopausal women. Although there are several studies regarding the risk of AD and vascular dementia after using MHT (Savolainen-Peltonen et al., 2019; Vinogradova et al., 2021), the present study’s findings are notable because they considered vascular dementia, which may be anticipated to occur at elevated rates in the presence of increased cerebral thrombosis risk in MHT users. This result is consistent with the finding of an earlier study that showed an overall increase of 20–40% in the risk of ischemic stroke.

The strength of our study was that it was a large-scale Asian population retrospective cohort study with a sufficient number of participants (*N* = 2,506,272) compared with the current observational study. It included many TIB users, and TIB is widely used in Europe, Asia, and Korea (Huang et al., 2010). Although we conducted a large-scale study of MHT effects on dementia, several issues limit the generalizability of our findings: the patients in our data were only Korean. Therefore, the generalization of the results to other ethnic or racial groups should be made with caution. There may have been detection bias in this study. Moreover, we did not have additional biomarkers indicating AD pathology, such as cerebrospinal fluid biomarkers, genetic testing, molecular imaging or neuropathological data from the participants. Also, although age is regarded to be an important parameter in estimating the risk of AD, our study has a limitation in that it did not reconcile younger skew of age and older age for development of AD. We assumed that non-AD dementia represents overall vascular dementia; however, other etiologies, such as Parkinson’s disease, may occur alongside non-AD dementia. Therefore, it is impossible to confirm the diagnosis code for any dementia, and our analysis may have led to a more inaccurate diagnosis. We assumed that most patients adhered to their physicians’ instructions as this study’s information on drug prescriptions was derived from claims data. However, menopausal women may not have adhered to their MHT prescriptions, which could have potentially introduced biases. It is also important to note that many Koreans prefer phytoestrogens over MHT (Kim Y. et al., 2022). Finally, although we analyzed as much data as possible, more information was needed regarding laboratory measurements, including serum hormone levels and blood pressure, which could be potential confounders.

This investigation showed that MHT had different effects on the incidence of AD and non-AD dementia, suggesting that it specifically increases the risk of non-AD dementia but that transdermal estrogen is not associated with dementia risk. The different effects of MHT should be considered in appropriately selected patients according to individual situations, such as underlying cardiovascular risk status.

## Data availability statement

The data analyzed in this study was obtained from The National Health Information Database of the National Health Insurance Service in South Korea. The datasets are not readily available because, due to NHIS’s privacy policy, only researchers have access to the data for a limited period of time. Further inquiries can be directed to the corresponding author.

## Ethics statement

The studies involving humans were approved by Institutional Review Board of the Sanggye Paik Hospital (approval number: SGPAIK-2020-08-002). The studies were conducted in accordance with the local legislation and institutional requirements. The participants provided their written informed consent to participate in this study.

## Author contributions

J-SY and JP: conception and design of the study, acquisition of data, and final approval of the manuscript. J-SY, JL, and JP: analysis and interpretation of the data, drafting and revising the manuscript for content. All authors contributed to the article and approved the submitted version.

## Conflict of interest

The authors declare that the research was conducted in the absence of any commercial or financial relationships that could be construed as a potential conflict of interest.

## Publisher’s note

All claims expressed in this article are solely those of the authors and do not necessarily represent those of their affiliated organizations, or those of the publisher, the editors and the reviewers. Any product that may be evaluated in this article, or claim that may be made by its manufacturer, is not guaranteed or endorsed by the publisher.
