# Supplementary material for: Menopausal hormone therapy and risk of dementia: health insurance database in South Korea-based retrospective cohort study
Source: Front Aging Neurosci. 2023 Sep 7;15:1213481. doi: 10.3389/fnagi.2023.1213481 (PMC10512830; doi:10.3389/fnagi.2023.1213481)
Supplement: Supplementary file 1 [file Data_Sheet_1.PDF]

Supplementary Material

Menopausal hormone therapy and risk of dementia: Health Insurance Database in South Korea-based retrospective cohort study

Jin-Sung Yuk[1], Jin San Lee[2], and Joong Hyun Park[3*]

[1]Department of Obstetrics and Gynecology, Sanggye Paik Hospital, School of Medicine, Inje University, Seoul, Republic of Korea

[2]Department of Neurology, Kyung Hee University Hospital, Kyung Hee University College of Medicine, Seoul, Republic of Korea

[3]Department of Neurology, Sanggye Paik Hospital, School of Medicine, Inje University, Seoul, Republic of Korea

*** Correspondence:**
Joong Hyun Park, MD, PhD
truelove1@hanmail.net

Supplementary table 1. A detailed list of menopausal hormones (generic and brand name)

MHT (generic name)	Brand name
**TIB**	
**Tibolone 2.5mg**	Livial, Rabilone, Libolone, Libron, Live on, Liviem, Tiborisi, Tibolan, Tibiol, Pharmbio Korea Tibolone, Hyundai Tibolone
**CEPM**	
**Estradiol Hemihydrate 1.03mg, Drospirenone 2mg**	Angelic
**Estradiol Valerate 2mg, Medroxyprogesterone Acetate 10mg**	Divina
**Estradiol Valerate 1mg, Medroxyprogesterone Acetate 2.5mg**	Indivina Tab 1mg/2.5mg
**Estradiol Valerate 1mg, Medroxyprogesterone Acetate 5mg**	Indivina Tab 1mg/5mg
**Estradiol Valerate 2mg, Medroxyprogesterone Acetate 2.5mg**	Indivina Tab 2mg/2.5mg
**Estradiol Hemihydrate 2mg, Norethisterone Acetate 1mg**	Cliane
**Estradiol Hemihydrate 2.06mg, Dydrogesterone 10mg**	Femoston 2/10
**Estradiol Hemihydrate 1.03mg, Dydrogesterone 10mg**	Femoston 1/10
**Estradiol Hemihydrate 1.03mg, Dydrogesterone 5mg**	Femoston Conti
**Cyproterone Acetate 1mg, Estradiol Valerate 2mg**	Climen
**Estradiol Hemihydrate 1.03mg, Norethisterone Acetate 0.5mg**	Esdiol-half
**Estradiol Valerate 1.31mg, Norethisterone Acetate 0.5mg**	Cliovelle
**Estrogen**	
**Conjugated Estrogens 0.3mg**	Premina 0.3mg
**Conjugated Estrogens 0.625mg**	Premina 0.625mg
**Estradiol Valerate 1mg**	Progynova 1mg
**Estradiol Valerate 2mg**	Progynova 2mg
**Estradiol Hemihydrate 1mg**	Preda 1mg
**CEPP**[a]	
**Progesterone Micronized 100mg**	Utrogestan 100mg
**Medroxyprogesterone Acetate 5mg**	Provera 5mg
**Medroxyprogesterone Acetate 10mg**	Provera 10mg
**Dydrogesterone 10mg**	Duphaston
**Transdermal estrogen**	
**Estradiol Hemihydrate, Estradiol**	Estreva Gel, Climara patch, Divigel Gel

MHT, menopausal hormone therapy; TIB, Tibolone; CEPM, Combined estrogen plus progestin by manufacturer; CEPP, Combined estrogen plus progestin by physician.
[a]This group used the progestin below and the estrogen group above simultaneously.

Supplementary table 2. Association of reproductive factors and dementia risk in women of MHT

	AD dementia[a]		Non-AD dementia[a]		Total dementia[a]	
**Variables**	HR (95% CI) [a]	*P*-value	HR (95% CI) [a]	*P*-value	HR (95% CI) [a]	*P*-value
**MHT**						
**TIB**	1.041 (1.01-1.072)	0.009	1.335 (1.303-1.368)	<0.001	1.212 (1.188-1.236)	<0.001
**CEPM**	0.975 (0.933-1.019)	0.266	1.25 (1.209-1.292)	<0.001	1.137 (1.106-1.169)	<0.001
**Oral Estrogen**	1.081 (1.03-1.134)	0.002	1.128 (1.079-1.179)	<0.001	1.092 (1.054-1.13)	<0.001
**CEPP**	1.131 (0.997-1.283)	0.055	1.046 (0.925-1.183)	0.469	1.087 (0.991-1.193)	0.077
**Transdermal estrogen**	0.989 (0.757-1.292)	0.937	0.919 (0.722-1.17)	1.493	0.892 (0.736-1.083)	0.249
**Age at inclusion (years)**						
**50~59**	3.471 (3.159-3.814)	<0.001	1.961 (1.869-2.058)	<0.001	2.23 (2.136-2.329)	<0.001
**60~69**	13.181 (11.929-14.565)	<0.001	3.565 (3.362-3.78)	<0.001	5.353 (5.092-5.627)	<0.001
**70~**	46.048 (41.603-50.967)	<0.001	6.767 (6.357-7.204)	<0.001	14.992 (14.23-15.794)	<0.001
**BMI (kg/m2)**						
**<18.5**	1.295 (1.234-1.358)	<0.001	1.026 (0.965-1.09)	0.414	1.184 (1.136-1.233)	<0.001
**23-24.9**	0.936 (0.917-0.956)	<0.001	1.023 (1.001-1.045)	0.038	0.983 (0.968-0.999)	0.033
**25-29.9**	0.91 (0.893-0.928)	<0.001	1.009 (0.989-1.03)	0.38	0.965 (0.951-0.98)	<0.001
**≥30**	0.908 (0.873-0.944)	<0.001	0.97 (0.929-1.012)	0.159	0.941 (0.912-0.97)	<0.001
**SES**						
**Low SES**	1.741 (1.681-1.803)	<0.001	1.96 (1.888-2.034)	<0.001	1.843 (1.793-1.895)	<0.001
**Region**						
**Rural area**	1.221 (1.198-1.244)	<0.001	1.131 (1.11-1.153)	<0.001	1.168 (1.152-1.185)	<0.001
**CCI**						
**1**	1.214 (1.191-1.236)	<0.001	1.141 (1.119-1.164)	<0.001	1.184 (1.167-1.202)	<0.001
**≥2**	1.288 (1.261-1.316)	<0.001	1.172 (1.145-1.199)	<0.001	1.238 (1.218-1.259)	<0.001
**Parity (years)**						
**0**	1.201 (1.142-1.263)	<0.001	0.993 (0.949-1.039)	0.758	1.07 (1.033-1.109)	<0.001
**2**	1.113 (1.063-1.165)	<0.001	1.028 (0.988-1.069)	0.176	1.066 (1.033-1.1)	<0.001
**≥3**	1.257 (1.197-1.32)	<0.001	1.043 (0.998-1.09)	0.06	1.116 (1.078-1.155)	<0.001
**Age at menarche (years)**						
**≥13**	0.953 (0.926-0.98)	<0.001	0.905 (0.879-0.932)	<0.001	0.915 (0.896-0.936)	<0.001
**Age at menopause (years)**						
**45-49**	1.03 (1.001-1.06)	0.043	1.13 (1.096-1.165)	<0.001	1.084 (1.06-1.108)	<0.001
**50-54**	1.099 (1.069-1.13)	<0.001	1.218 (1.18-1.257)	<0.001	1.161 (1.136-1.188)	<0.001
**55-**	1.187 (1.143-1.233)	<0.001	1.415 (1.356-1.476)	<0.001	1.288 (1.25-1.328)	<0.001
**Smoking**						
**Past**	1.249 (1.16-1.344)	<0.001	1.165 (1.081-1.255)	<0.001	1.212 (1.146-1.281)	<0.001
**Current**	1.292 (1.234-1.353)	<0.001	1.172 (1.118-1.229)	<0.001	1.239 (1.197-1.284)	<0.001
**Alcohol (g/week)**						
**~2/week**	0.878 (0.852-0.905)	<0.001	1.004 (0.978-1.032)	0.751	0.951 (0.931-0.971)	<0.001
**3~6/week**	0.923 (0.847-1.007)	0.071	1.11 (1.034-1.191)	0.004	1.044 (0.986-1.106)	0.139
**Daily**	1.021 (0.92-1.134)	0.694	1.152 (1.041-1.274)	0.006	1.084 (1.003-1.171)	0.043
**Physical exercise (per week)**						
**1~2**	0.844 (0.824-0.865)	<0.001	0.899 (0.878-0.921)	<0.001	0.867 (0.851-0.883)	<0.001
**3~4**	0.781 (0.756-0.807)	<0.001	0.868 (0.841-0.895)	<0.001	0.825 (0.806-0.845)	<0.001
**5~6**	0.791 (0.749-0.835)	<0.001	0.92 (0.875-0.967)	0.001	0.862 (0.829-0.896)	<0.001
**Daily**	0.955 (0.927-0.983)	0.002	0.974 (0.944-1.005)	0.101	0.957 (0.935-0.979)	<0.001
**Period from menopause to inclusion (years)**						
**5~9**	1.581 (1.516-1.648)	<0.001	1.391 (1.352-1.432)	<0.001	1.413 (1.379-1.448)	<0.001
**10~**	2.783 (2.66-2.911)	<0.001	1.926 (1.857-1.997)	<0.001	2.165 (2.103-2.228)	<0.001

MHT, menopausal hormone therapy; AD, Alzheimer's disease; HR, hazard ratio; CI, confidence interval; TIB, Tibolone; CEPM, Combined estrogen plus progestin by manufacturer; CEPP, Combined estrogen plus progestin by physician; BMI, Body mass index; CCI, Charlson comorbidity index; SES, socioeconomic status.

[a]HRs were adjusted for age group, BMI, SES region, CCI, parity, age at menarche, age at menopause, smoking, alcohol, physical exercise, and period from menopause to inclusion.

Supplementary table 3. Case/person-years of dementia in Korea National Health Insurance Data, 2002-2019

	Non-MHT	TIB	CEPM	Oral Estrogen	CEPP	Transdermal estrogen
**Total**	96,853/11,494,291 (843)	13,372/2,457,317 (544)	6,183/1,577,476 (392)	3,696/705,854 (524)	494/86,555 (571)	115/28,317 (406)
**Age at inclusion (years)**						
**40~49**	960/1,126,771 (85)	757/447,153 (169)	538/369,975 (145)	265/193,333 (137)	28/15,817 (177)	4/6,297 (64)
**50~59**	15,742/5,748,549 (274)	6,489/1,611,114 (403)	3,496/1,042,616 (335)	1,374/394,763 (348)	168/51,625 (325)	53/17,347 (306)
**60~69**	40,843/3,452,219 (1183)	4,818/362,572 (1329)	1,795/154,264 (1164)	1,457/100,597 (1448)	231/17,108 (1350)	46/4,292 (1072)
**70~**	39,308/1,166,752 (3369)	1,308/36,478 (3586)	354/10,621 (3333)	600/17,161 (3496)	67/2,005 (3342)	12/381 (3147)
**BMI (kg/m2)**						
**<18.5**	2,256/201,226 (1121)	211/40,784 (517)	106/30,490 (348)	63/10,012 (629)	10/1,754 (570)	7/549 (1275)
**18.5-22.9**	28,254/3,870,257 (730)	4,382/976,520 (449)	2,222/701,457 (317)	1,178/257,286 (458)	159/35,587 (447)	33/10,469 (315)
**23-24.9**	23,649/3,023,316 (782)	3,698/680,970 (543)	1,719/424,716 (405)	960/197,175 (487)	140/24,262 (577)	34/7,569 (449)
**25-29.9**	32,654/3,755,281 (870)	4,237/677,852 (625)	1,810/380,023 (476)	1,273/212,846 (598)	159/22,548 (705)	37/8,755 (423)
**≥30**	4,183/481,581 (869)	420/61,365 (684)	177/31,048 (570)	129/23,916 (539)	12/1,862 (644)	1/791 (126)
**SES**						
**Mid~high SES**	86,602/11,096,322 (780)	12,267/2,380,190 (515)	5,790/1,541,666 (376)	3,467/689,153 (503)	466/84,743 (550)	109/27,603 (395)
**Low SES**	10,251/397,968 (2576)	1,105/77,127 (1433)	393/35,810 (1097)	229/16,701 (1371)	28/1,812 (1545)	6/714 (840)
**Region**						
**Urban area**	22,426/3,419,846 (656)	4,080/772,984 (528)	2,085/538,692 (387)	1,011/224,957 (449)	221/44,248 (499)	51/12,860 (397)
**Rural area**	74,427/8,074,444 (922)	9,292/1,684,333 (552)	4,098/1,038,784 (394)	2,685/480,897 (558)	273/42,306 (645)	64/15,457 (414)
**CCI**						
**0**	55,403/7,643,636 (725)	8,059/1,677,987 (480)	3,978/1,120,359 (355)	2,270/494,421 (459)	289/60,214 (480)	68/18,666 (364)
**1**	24,278/2,225,501 (1091)	3,096/477,204 (649)	1,341/285,020 (470)	854/127,772 (668)	126/16,074 (784)	30/5,199 (577)
**≥2**	17,172/1,625,154 (1057)	2,217/302,127 (734)	864/172,097 (502)	572/83,661 (684)	79/10,267 (769)	17/4,452 (382)
**Parity (years)**						

**0 or not respond**	18,092/2,322,929 (779)	2,632/430,151 (612)	1,099/230,173 (477)	784/159,797 (491)	123/18,702 (658)	29/6,427 (451)
**1**	3,596/640,030 (562)	818/203,712 (402)	472/155,569 (303)	177/52,433 (338)	33/6,059 (545)	6/2,170 (276)
**2**	58,322/6,931,124 (841)	8,131/1,585,265 (513)	3,894/1,067,781 (365)	2,170/420,807 (516)	264/52,513 (503)	69/16,727 (413)
**≥3**	16,843/1,600,208 (1053)	1,791/238,188 (752)	718/123,953 (579)	565/72,817 (776)	74/9,280 (797)	11/2,993 (368)
**Age at menarche (years)**						
**<13**	11,942/2,147,233 (556)	1,893/376,306 (503)	885/235,760 (375)	601/134,231 (448)	83/16,726 (496)	25/5,331 (469)
**≥13**	84,033/9,278,588 (906)	11,311/2,059,612 (549)	5,241/1,331,232 (394)	3,045/562,041 (542)	399/69,071 (578)	89/22,625 (393)
**Age at menopause (years)**						
**40-44**	10,198/1,592,617 (640)	1,418/314,531 (451)	631/191,354 (330)	526/157,459 (334)	48/11,726 (409)	19/5,781 (329)
**45-49**	28,159/3,357,951 (839)	4,082/812,168 (503)	1,912/533,609 (358)	1,231/252,216 (488)	152/28,349 (536)	40/10,310 (388)
**50-54**	48,937/5,590,750 (875)	6,352/1,147,837 (553)	3,002/747,809 (401)	1,661/262,482 (633)	238/39,765 (599)	44/10,484 (420)
**55-**	9,559/952,973 (1003)	1,520/182,782 (832)	638/104,704 (609)	278/33,697 (825)	56/6,715 (834)	12/1,741 (689)
**Smoking**						
**Never**	82,953/10,554,898 (786)	11,676/2,221,262 (526)	5,453/1,428,125 (382)	3,294/645,767 (510)	438/80,008 (547)	101/25,967 (389)
**Past**	946/106,246 (890)	192/39,560 (485)	109/27,215 (401)	51/9,210 (554)	7/1,037 (675)	3/416 (721)
**Current**	2,442/270,156 (904)	528/104,887 (503)	264/70,295 (376)	116/24,395 (476)	18/2,381 (756)	1/627 (159)
**Alcohol (g/week)**						
**None**	78,421/9,344,469 (839)	10,328/1,852,354 (558)	4,679/1,169,205 (400)	3,015/546,510 (552)	405/69,695 (581)	94/22,525 (417)
**~2/week**	6,845/1,413,974 (484)	1,794/454,008 (395)	997/315,235 (316)	387/121,162 (319)	57/12,786 (446)	12/4,337 (277)
**3~6/week**	787/161,030 (489)	247/58,429 (423)	149/40,625 (367)	52/12,511 (416)	2/1,139 (176)	1/409 (245)
**Daily**	497/62,593 (794)	101/19,171 (527)	41/11,184 (367)	20/5,110 (391)	/546 (0)	1/129 (774)
**Physical exercise (per week)**						
**None**	61,712/7,020,391 (879)	7,690/1,391,927 (552)	3,550/904,542 (392)	2,292/402,707 (569)	271/47,271 (573)	59/14,198 (416)
**1~2**	10,875/1,882,383 (578)	2,089/462,046 (452)	1,003/303,777 (330)	520/133,582 (389)	76/16,642 (457)	22/5,969 (369)
**3~4**	5,642/1,052,865 (536)	1,263/279,816 (451)	627/183,212 (342)	292/77,528 (377)	57/11,049 (516)	16/4,025 (397)
**5~6**	2,035/343,656 (592)	423/90,813 (466)	230/59,543 (386)	94/24,170 (389)	22/3,291 (668)	4/1,177 (340)

**Daily**	6,465/699,292 (925)	1,004/157,073 (639)	457/84,806 (539)	287/46,722 (614)	37/5,831 (635)	6/1,914 (314)
**Period from menopause to inclusion (years)**						
**<5**	9,247/4,597,657 (201)	4,278/1,421,016 (301)	2,549/1,060,103 (240)	882/375,879 (235)	113/44,622 (253)	27/14,679 (184)
**5~9**	11,956/2,571,664 (465)	3,110/581,051 (535)	1,447/324,295 (446)	715/177,693 (402)	96/21,355 (450)	27/7,437 (363)
**10~**	75,650/4,324,970 (1749)	5,984/455,250 (1314)	2,187/193,078 (1133)	2,099/152,283 (1378)	285/20,578 (1385)	61/6,201 (984)

TIB, Tibolone; CEPM, Combined estrogen plus progestin by manufacturer; CEPP, Combined estrogen plus progestin by physician; BMI, Body mass index; CCI, Charlson comorbidity index; MHT, menopausal hormone therapy; SES, socioeconomic status.

Data are expressed as the case/person-years (case/ 100,000 person-years).
